# Supplementary material for: Strategies to improve sexual health in women with urinary incontinence: A scoping review
Source: PLoS One. 2026 Apr 17;21(4):e0346256. doi: 10.1371/journal.pone.0346256 (PMC13089752; doi:10.1371/journal.pone.0346256)
Supplement: S1 File — (Characteristics of included studies, study design, population, intervention type, sexual health outcomes, and key findings). (DOCX) [file pone.0346256.s001.docx]

| Supplementary File 1. Key Features of Studies Included in the Scoping Review | | | | | | | | | | |
| --- | --- | --- | --- | --- | --- | --- | --- | --- | --- | --- |
| Number | Authors | Population | Type of Incontinence | Intervention and Outcomes | Design | Control Group Information | Outcome Measures and Tools Used | Follow-up Period | Results | Place |
| 1 | Ko et al. (2015) [29] | 65 sexually active women with stress urinary incontinence (SUI) undergoing transobturator tape (TOT) procedure and their male partners | Stress Urinary Incontinence (SUI) | To evaluate the effect of TOT procedure on sexual function of patients and their spouses over a 12-month period. | Prospective single-center study using FSFI for women and MSHQ for male partners | Not applicable (prospective single-center study without a control group) | FSFI for women, MSHQ for male partners | 12 months | Significant improvement in sexual function for women; male partners reported no significant change in sexual satisfaction. | South Korea; single-center study. |
| 2 | Glavind et al. (2015) [36] | 81 sexually active women with pelvic organ prolapse (POP) stage ≥2 undergoing native tissue repair | Pelvic Organ Prolapse (POP), Stress Urinary Incontinence (SUI) | To evaluate sexual function before and after native tissue repair for POP and its association with urinary incontinence. | Prospective observational study using PISQ-12-SF and ICIQ-UI-SF questionnaires | Not applicable (prospective observational study without a control group) | PISQ-12-SF, ICIQ-UI-SF questionnaires | 6 months | Improvement in urinary symptoms and sexual function post native tissue repair; significant reduction in coital incontinence. | Denmark; single-center study. |
| 3 | Surkont et al. (2015) [35] | 227 women with stress urinary incontinence (SUI) undergoing modified colposuspension | Stress Urinary Incontinence (SUI) | To evaluate mid-term effects and patient satisfaction with standardized modified colposuspension. | Prospective study with mid-term follow-up (9–36 months) using VAS and clinical assessments | Not applicable (prospective study without a control group) | VAS, clinical assessments for POP and urinary symptoms | 9–36 months (mid-term follow-up) | High patient satisfaction and significant improvement in urinary and sexual symptoms; 85% reported no incontinence post-colposuspension. | Poland; single-center study. |
| 4 | Meriwether et al. (2015) [28] | 127 women using pessaries for pelvic floor disorders | Pelvic Organ Prolapse (POP), Stress Urinary Incontinence (SUI) | To describe sexual function and pessary management regarding sexual activity in women using pessaries. | Secondary analysis of a randomized trial using PISQ-IR and mBIS questionnaires with a 3-month follow-up | Yes, randomized control groups (horizontal vs. vertical vaginal cuff closure) | PISQ-12 questionnaire for sexual function assessment | 6 months | No significant difference in sexual function between horizontal and vertical cuff closure groups; overall improvement in urinary symptoms post-VH. | Turkey; multi-center study. |
| 5 | Lukacz et al. (2016) [49] | 374 women with apical pelvic organ prolapse (POP) undergoing native tissue vaginal apical prolapse repair with anti-incontinence surgery | Pelvic Organ Prolapse (POP), Stress Urinary Incontinence (SUI) | To assess the impact of native tissue vaginal apical prolapse repair on quality of life, sexual function, and body image over a 24-month follow-up. | Multicenter randomized trial using PFDI, PFIQ, SF-36, and PISQ-12 questionnaires at baseline, 6, 12, and 24 months | Not applicable (prospective study without a control group) | IIQ-7, UDI-6, FSFI for patients; IIEF-5 for spouses | 6 months | Improvement in sexual function for patients post-TOT; no significant change in sexual function for spouses. | Turkey; single-center study. |
| 6 | Detollenaere et al. (2016) [41] | 208 women with uterine prolapse stage II or higher undergoing sacrospinous hysteropexy or vaginal hysterectomy | Pelvic Organ Prolapse (POP), Stress Urinary Incontinence (SUI) | To compare sexual function after sacrospinous hysteropexy and vaginal hysterectomy with uterosacral ligament suspension in women with uterine prolapse. | Secondary analysis of a multicenter randomized trial (SAVE U trial) using PISQ-12 questionnaire | Yes, comparison between a-TOT, TOT, and RT groups | FSFI, PGI-I scales | 12 months | All groups showed improvement in sexual function post-surgery; a-TOT group reported higher satisfaction and fewer complications. | Turkey; multi-center study. |
| 7 | Mengerink et al. (2016) [30] | 578 women with stress urinary incontinence (SUI) undergoing midurethral sling (MUS) surgery | Stress Urinary Incontinence (SUI) | To assess the impact of midurethral sling (MUS) surgery on sexual activity and function, including coital incontinence and dyspareunia. | Secondary analysis of a non-inferiority randomized controlled trial (VUSIS-II) using validated and non-validated questionnaires | Not applicable (historical cohort study without a control group) | Norwegian Female Incontinence Registry, validated questionnaires | 10–20 years | Long-term improvement in sexual function and reduced impact of incontinence on sexual life post-MUS surgery. | Norway; data collected from a national registry. |
| 8 | Lindquist et al. (2016) [31] | 63 women with stress urinary incontinence (SUI) undergoing tension-free vaginal tape (TVT) surgery | Stress Urinary Incontinence (SUI) | To assess long-term sexual function before and after TVT surgery and compare short-term and long-term outcomes. | Prospective study using PISQ-12 and ICIQ-UI SF questionnaires with preoperative, 6-month, and long-term follow-ups (mean 4 years and 9 months) | Not applicable (prospective study without a control group) | ePAQ-PF questionnaire for preoperative and 6-month postoperative assessment | 6 months | Laparoscopic Sacrocolpopexy (LSC) significantly improved vaginal, urinary, bowel, and sexual symptoms at 6 months. | Greece; single-center study. |
| 9 | Selcuk et al. (2016) [42] | 142 women undergoing simple (Type I) or radical (Type III) hysterectomy for benign or malignant conditions, and a control group with no surgery | Pelvic Organ Prolapse (POP), Stress Urinary Incontinence (SUI) | To evaluate the impact of simple and radical hysterectomy on pelvic floor dysfunctions including urinary and sexual dysfunction. | Retrospective cohort study using UDI-6, IIQ-7, PISQ-12, and Wexner Incontinence Scale | Not applicable (prospective non-randomized study without a control group) | ICIQ-UI, BFLUTS-SF, PFDI-20, PISQ-12 questionnaires | 12 months | Different hysterectomy types have varying impacts, with Type I linked to worse urinary symptoms and Type III to poorer sexual function | Israel; single-center study. |
| 10 | Siff et al. (2016) [33] | 526 women with stress urinary incontinence (SUI) undergoing midurethral sling (MUS) surgery; 79 with major depression | Stress Urinary Incontinence (SUI) | To determine the impact of major depression on urinary incontinence severity, quality of life, and sexual function after MUS surgery. | Secondary analysis of the TOMUS trial using PHQ-9, ICIQ, IIQ, UDI, and PISQ-12 with a 12-month follow-up | Not applicable (interventional prospective study without a control group) | PISQ-12, ICIQ-SF, I-QOL questionnaires | 24 months | Adjustable tension-free suburethral mesh system significantly improved sexual function and quality of life; no severe complications reported. | Spain; single-center study. |
| 11 | Uçar et al. (2016) [43] | 78 women with uterine prolapse stage 2 or higher; underwent vaginal hysterectomy (VH) | Pelvic Organ Prolapse (POP) | To evaluate sexual function before and after VH and compare effects of horizontal vs. vertical vaginal cuff closure. | Prospective, randomized study using PISQ-12 questionnaire | Yes, comparison between sacrospinous hysteropexy and vaginal hysterectomy groups | PISQ-12 questionnaire | 12 months | Both surgical methods improved sexual function; sacrospinous hysteropexy had a lower complication rate and faster recovery. | The Netherlands; multi-center study (SAVE U trial). |
| 12 | Jha et al. (2017) [23] | 84 sexually active women with urinary incontinence (mean age 45.6 ± 12.3 years) | Stress, Urge, and Mixed UI | To compare the effectiveness of PFMT plus electrical stimulation vs. PFMT alone on sexual function | Randomized Controlled Trial (Two-arm, single-center, parallel-group) | Standard PFMT group (control) vs. PFMT + Electrical Stimulation (intervention) | PISQ-12 (Pelvic Organ Prolapse/Urinary Incontinence Sexual Questionnaire), SF-36, EQ-5D, ePAQ-PF | 12 weeks | Both groups showed improvement in sexual function; no statistically significant difference between groups; electrical stimulation was cost-effective | Sheffield, UK |
| 13 | Schiavi et al. (2017) [37] | 146 women with severe pelvic organ prolapse (POP) stage ≥III undergoing vaginal native tissue repair (VNTR) | Pelvic Organ Prolapse (POP), Stress Urinary Incontinence (SUI) | To assess the effectiveness and safety of VNTR for POP and its impact on sexual function and quality of life over a median follow-up of 48 months. | Retrospective cohort study using ICIQ-UI SF, P-QoL, and PISQ-12 questionnaires | Yes, comparison between transobturator sling, retropubic sling, Burch procedure, and fascial sling groups | PISQ-12 questionnaire | 24 months | All surgical methods significantly improved sexual function; fascial sling group had higher rates of postoperative dyspareunia. | United States; multi-center study. |
| 14 | Leshem et al. (2017) [45] | 150 obese women with pelvic floor disorders undergoing bariatric surgery | Stress Urinary Incontinence (SUI), Mixed Urinary Incontinence (MUI), Urgency Urinary Incontinence (UUI) | To assess the effect of bariatric surgery-induced weight loss on urinary incontinence, pelvic organ prolapse, colorectal-anal symptoms, and sexual dysfunction. | Prospective, non-randomized study using ICIQ-UI, BFLUTS-SF, PFDI-20, PISQ-12 questionnaires | Not applicable (prospective study without a control group) | GRISS, ICIQ-UI SF questionnaires | 6 months | Weight loss surgery significantly improves pelvic floor disorders and sexual performance. | South Korea; single-center study. |
| 15 | Lim et al. (2017) [25] | 66 couples with female partners diagnosed with stress urinary incontinence (SUI) receiving pulsed magnetic stimulation (PMS) | Stress Urinary Incontinence (SUI) | To assess the effect of pulsed magnetic stimulation (PMS) on sexual function in couples with female SUI partners. | Prospective study using GRISS and ICIQ-UI SF questionnaires with pre-treatment, post-treatment, and 6-month follow-up assessments | Not applicable (prospective study without a control group) | PISQ-12, ICIQ-UI SF questionnaires | Preoperative, 6 months, and long-term (mean 4 years 9 months) | Long-term improvement in sexual function and reduction in urinary incontinence symptoms post-TVT surgery. | Sweden; single-center study. |
| 16 | Koroglu et al. (2018) [44] | 182 women who underwent total abdominal hysterectomy (TAH) or total laparoscopic hysterectomy (TLH) for benign causes | Pelvic Organ Prolapse (POP) | To assess POP development and sexual function 1 year after hysterectomy, comparing TLH and TAH results. | Prospective cohort study at a tertiary hospital | Not applicable (cohort study without a control group) | Pelvic Organ Prolapse Quantification (POP-Q), PISQ-12 | 12 months | No significant differences in POP-Q measurements or PISQ-12 scores between TLH and TAH; postoperative vaginal length significantly shorter in TAH group, but no clinical impact on sexual function. | Turkey; single-center study. |
| 17 | Vitale et al. (2018) [51] | 20 sexually active women with second recurrence of vaginal vault prolapse (VVP) previously treated with monolateral sacrospinous fixation (SF) | Vaginal Vault Prolapse (VVP), Stress Urinary Incontinence (SUI) | To evaluate the efficacy and impact of transvaginal bilateral sacrospinous fixation (TBSF) on quality of life (QoL) and sexual functions in women with VVP. | Prospective observational study using POP-Q system, SF-36, and PISQ-12 questionnaires with a 12-month follow-up | Not applicable (prospective observational study without a control group) | POP-Q system, SF-36, PISQ-12 questionnaires | 12 months | Transvaginal bilateral sacrospinous fixation (TBSF) significantly improved sexual function and quality of life in women with recurrent vaginal vault prolapse. | Italy; single-center study. |
| 18 | Pérez-Tomás et al. (2018) [39] | 60 sexually active women with stress urinary incontinence (SUI) or mixed urinary incontinence (MUI) undergoing adjustable tension-free mesh system surgery | Stress Urinary Incontinence (SUI), Mixed Urinary Incontinence (MUI) | To assess the impact of adjustable tension-free suburethral mesh system (TOA/TVA) on sexual quality of life in sexually active women. | Interventional prospective study with a two-year follow-up using PISQ-12, ICIQ-SF, and I-QOL questionnaires | Not applicable (interventional prospective study without a control group) | PISQ-12, ICIQ-SF, I-QOL questionnaires | 24 months | Adjustable tension-free suburethral mesh system significantly improved sexual function and quality of life; no severe complications reported. | Spain; single-center study. |
| 19 | Hsiao et al. (2019) [52] | 205 women with symptomatic pelvic organ prolapse (POP) stages II to IV undergoing Uphold transvaginal mesh surgery | Pelvic Organ Prolapse (POP), Stress Urinary Incontinence (SUI) | To assess the effect of Uphold transvaginal mesh surgery on sexual function and anatomical restoration of POP. | Prospective cohort study using UDI-6, IIQ-7, and FSFI questionnaires with a 6-month follow-up | Not applicable (prospective cohort study without a control group) | UDI-6, IIQ-7, FSFI questionnaires | 6 months | Uphold transvaginal mesh surgery significantly improved sexual function and anatomical restoration of POP; low complication rates. | Taiwan; single-center study. |
| 20 | Apostolopoulos et al. (2019) [40] | 27 women with symptomatic post-hysterectomy vaginal vault prolapse undergoing Laparoscopic Sacrocolpopexy (LSC) | Stress Urinary Incontinence (SUI) associated with pelvic organ prolapse (POP) | To assess the early benefits of LSC on vaginal, urinary, bowel, and sexual symptoms at 6-month follow-up. | Prospective study using ePAQ-PF questionnaire for preoperative and 6-month postoperative assessment | Not applicable (prospective study without a control group) | ePAQ-PF questionnaire for preoperative and 6-month postoperative assessment | 6 months | Laparoscopic Sacrocolpopexy (LSC) significantly improved vaginal, urinary, bowel, and sexual symptoms at 6 months. | Greece; single-center study. |
| 21 | Emami et al. (2019) [46] | 80 women with stress urinary incontinence (SUI), divided into mini-sling and TOT groups (40 each) | Stress Urinary Incontinence (SUI) | To compare the efficacy, complications, urodynamic changes, and sexual function between mini-sling and transobturator tape (TOT) methods. | Randomized controlled trial using FSFI and ICIQ questionnaires | Yes, randomized comparison between mini-sling and TOT groups | FSFI, ICIQ questionnaires | 12 months | Both mini-sling and TOT improved sexual function and urinary symptoms; mini-sling group had fewer postoperative complications. | Iran; single-center study. |
| 22 | Grzybowska & Wydra (2019) [24] | 350 women with pelvic floor disorders (PFD); 173 sexually active (SA) and 177 not sexually active (NSA) | Pelvic Organ Prolapse (POP), Urinary Incontinence (UI), Mixed (POP and UI) | To investigate the relationships between pelvic floor muscles (PFM) parameters and sexual function (SF) in women with PFD. | Cross-sectional study using clinical evaluation and questionnaires (PISQ-IR, FSFI) | Not applicable (prospective study without a control group) | FSFI, ICIQ-SF, PISQ-12 questionnaires | 12 months | Significant improvement in sexual function and urinary symptoms post-treatment; no severe complications reported. | Poland; single-center study. |
| 23 | Glass Clark et al. (2020) [38] | Women with stress urinary incontinence (SUI) undergoing transobturator sling, retropubic sling, Burch procedure, or fascial sling surgeries | Stress Urinary Incontinence (SUI) | To assess the impact of different surgical interventions for SUI on female sexual function over 24 months. | Prospective cohort study using PISQ-12 questionnaire with follow-ups at 12 and 24 months | Yes, comparison between transobturator sling, retropubic sling, Burch procedure, and fascial sling groups | PISQ-12 questionnaire | 24 months | All surgical methods significantly improved sexual function; fascial sling group had higher rates of postoperative dyspareunia. | United States; multi-center study. |
| 24 | Maher et al. (2020) [48] | Women undergoing repeat anterior or posterior prolapse surgery using mesh inlay, mesh kit, or native tissue repair | Pelvic Organ Prolapse (POP), Stress Urinary Incontinence (SUI) | To compare outcomes of mesh inlay, mesh kit, and native tissue repair in repeat prolapse surgeries focusing on anatomical outcomes, quality of life, and reoperation rates. | Randomized controlled trial (PROSPECT) using validated outcome measures and follow-ups | Yes, comparison between mesh inlay, mesh kit, and native tissue repair groups | Validated outcome measures (not specified in detail) | 12 months | All three surgical methods significantly improved anatomical outcomes and quality of life; native tissue repair had fewer complications. | United Kingdom; multi-center study (PROSPECT trial). |
| 25 | Oğlak & Obut (2020) [50] | 157 women with stress urinary incontinence (SUI) and their spouses | Stress Urinary Incontinence (SUI) | To investigate the effects of transobturator tape (TOT) procedure on sexual function of patients and their spouses. | Prospective study using IIQ-7, UDI-6, FSFI for patients and IIEF-5 for spouses | Not applicable (prospective study without a control group) | IIQ-7, UDI-6, FSFI for patients; IIEF-5 for spouses | 6 months | Improvement in sexual function for patients post-TOT; no significant change in sexual function for spouses. | Turkey; single-center study. |
| 26 | Çubuk et al. (2021) [32] | 142 sexually active women with stress urinary incontinence (SUI) undergoing a-TOT, TOT, and RT surgeries | Stress Urinary Incontinence (SUI) | To compare the impact of autologous transobturator tape (a-TOT) with TOT and RT on female sexual function and incontinence outcomes. | Retrospective cohort study using FSFI and PGI-I scales | Yes, comparison between a-TOT, TOT, and RT groups | FSFI, PGI-I scales | 12 months | All groups showed improvement in sexual function post-surgery; a-TOT group reported higher satisfaction and fewer complications. | Turkey; multi-center study. |
| 27 | Atılgan & Eren (2021) [34] | 82 sexually active women with stress urinary incontinence (SUI) and coital incontinence (CI) | Stress Urinary Incontinence (SUI) and Coital Incontinence (CI) | To evaluate the impact of tension-free vaginal tape (TVT) on coital incontinence concomitant with SUI and its effect on sexual function. | Prospective observational study using FSFI and PGI-I scales | Not applicable (prospective observational study without a control group) | FSFI, PGI-I scales | 12 months | Significant improvement in coital incontinence and sexual function post-TVT; most patients reported improvement in overall condition. | Turkey; single-center study. |
| 28 | White et al. (2021) [47] | 281 women with stress urinary incontinence (SUI) undergoing single incision sling (SIS) or transobturator midurethral sling (TMUS) | Stress Urinary Incontinence (SUI) | To compare sexual function 36 months postoperatively between patients undergoing SIS and TMUS for SUI. | Prospective, non-randomized, parallel cohort, multi-center study across 21 sites in the United States and Australia. | Yes, comparison between SIS (Solyx) and TMUS (Obtryx II) | PISQ-12, Pelvic Floor Impact Questionnaire-7, Urogenital Distress Inventory, Incontinence Severity Index, PGI-I | 36 months | \| Significant improvement in sexual function postoperatively in both SIS and TMUS groups; minimal de novo dyspareunia; TMUS group showed slightly greater improvement at 36 months. \| \| --- \|  \|  \| \| --- \| | United States, Australia; multi-center study. |
| 29 | González-Isaza et al. (2022) [26] | 47 women aged 18–80 with stress urinary incontinence (SUI) undergoing pulsed magnetic stimulation (PMS) | Stress Urinary Incontinence (SUI) | To assess the efficacy and safety of pulsed magnetic stimulation (PMS) on urinary incontinence and its impact on sexual function and quality of life. | Randomized controlled trial using FSFI, ICQSF, and PFBQ questionnaires with follow-ups at 1, 9, and 14 weeks | Yes, randomized controlled trial with a control group | FSFI, ICQSF, PFBQ questionnaires | 1, 9, and 14 weeks | Pulsed magnetic stimulation (PMS) significantly improved urinary incontinence and sexual function; high safety and efficacy reported. | Colombia; single-center study. |
| 30 | Moradinasab et al. (2023) [27] | 84 reproductive-aged women (18 to 45 years old) with urinary incontinence | Stress Urinary Incontinence (SUI), Urgency Urinary Incontinence (UUI), Mixed Urinary Incontinence (MUI) | To investigate the effect of cognitive–behavioral therapy (CBT) on sexual self-esteem and sexual function in reproductive-aged women with urinary incontinence. | Randomized controlled clinical trial using ICIQ-SF, SSEL-W-SF, and PISQ-12 questionnaires | Yes, randomized controlled clinical trial with a control group | ICIQ-SF, SSEL-W-SF, PISQ-12 questionnaires | 12 weeks | Cognitive–behavioral therapy (CBT) significantly improved sexual self-esteem and sexual function in women with urinary incontinence. | Iran; single-center study. |
| 31 | Solhaug et al. (2024) [22] | 1210 women with stress urinary incontinence (SUI) who underwent mid-urethral sling (MUS) surgery | Stress Urinary Incontinence (SUI) | To assess long-term sexual function and impact of incontinence on sexual life 10–20 years after MUS surgery. | Historical cohort study using the Norwegian Female Incontinence Registry and validated questionnaires | Not applicable (historical cohort study without a control group) | Norwegian Female Incontinence Registry, validated questionnaires | 10–20 years | Long-term improvement in sexual function and reduced impact of incontinence on sexual life post-MUS surgery. | Norway; data collected from a national registry. |
