# Supplementary material for: Strategies to improve sexual health in women with urinary incontinence: A scoping review
Source: PLoS One. 2026 Apr 17;21(4):e0346256. doi: 10.1371/journal.pone.0346256 (PMC13089752; doi:10.1371/journal.pone.0346256)
Supplement: Attachment — Submitted filename: point by point askarloo.docx [file pone.0346256.s004.docx]

Dear Dr. Lee,
Dear Reviewers,

We would like to thank the Academic Editor and both reviewers for their careful evaluation of our manuscript entitled “Strategies to improve sexual health in women with urinary incontinence: A scoping review” (PONE-D-25-66253). We appreciate the constructive and insightful comments, which have helped us substantially improve the methodological transparency, conceptual clarity, and clinical interpretation of our review.

Below, we respond point by point to each comment. Reviewer comments are reproduced verbatim in italics, followed by our responses and a description of the revisions made.

**Responses to Reviewer #1**

**Comment 1**

*“Only the PubMed search is clearly shown… PRISMA-ScR requires that at least one full search strategy be shown and that the others be clearly explained… some sexual health terms are missing (dyspareunia, vaginal pain, libido, arousal, sexual desire).”*

**Response:**
We thank the reviewer for highlighting this important methodological issue. We have now fully expanded the search strategy to ensure transparency and reproducibility in accordance with PRISMA-ScR guidelines.

- Full, reproducible search strings for PubMed, Scopus, Web of Science, and ScienceDirect are provided in Table1.
- We expanded the sexual health–related keywords to include dyspareunia, vaginal pain, libido, arousal, sexual desire, and related synonyms.
- The Methods section has been revised to clearly state how each database was searched and how terminology was adapted across platforms.

*Sexual health–related terms were intentionally broadened to include dyspareunia, libido, sexual desire, arousal, and vaginal pain to minimize the risk of missing relevant studies*

**Comment 2**

*“Limiting the search to the last 10 years leaves out older but important PFMT and MUS studies… Please explain why these older studies were excluded.”*

**Response**:
We appreciate this observation. We have now explicitly justified the time restriction in the Methods section. The rationale for limiting the search to the last 10 years was to:

1. Capture contemporary surgical techniques (e.g., modern mid-urethral sling modifications),
2. Reflect current PFMT protocols, and
3. Include recently validated sexual function instruments and scoring interpretations.

To address the reviewer’s concern, we now:

- Emphasize that this scoping review maps recent evidence trends, not the historical evolution of treatments.

*The literature search was limited to studies published between January 2015 and December 2025. This timeframe was selected to capture evidence reflecting contemporary clinical practice in the management of urinary incontinence, including modern surgical techniques (e.g., current mid-urethral sling approaches), updated pelvic floor muscle training (PFMT) protocols, and the widespread use of validated sexual function instruments such as the FSFI and PISQ-12 with standardized scoring and interpretation. Given substantial changes in intervention techniques and outcome measurement over the past decade, restricting the timeframe was intended to enhance the clinical relevance and interpretability of the findings*.

**Comment 3**

*“The PRISMA flow diagram has inconsistent numbers… The entire diagram needs to be redone.”*

**Response:**
We agree and thank the reviewer for identifying these inconsistencies. The PRISMA-ScR flow diagram has been completely redrawn.

**Comment 4**

*“You used MMAT and excluded studies scoring below 3/5. Scoping reviews usually do not exclude studies based on quality.”*

**Response:**
We thank the reviewer for this important methodological comment and fully acknowledge that scoping reviews typically do not exclude studies based on methodological quality. In this review, however, the application of a quality threshold served a specific preparatory purpose rather than a traditional evidence-synthesis aim.

The present scoping review was designed as a preliminary evidence-mapping step to inform the future development of a sexual health guideline for women with urinary incontinence. Given this applied objective, we prioritized studies with sufficient methodological rigor to ensure that the strategies identified would be based on reliable and interpretable evidence, suitable for translation into clinical or educational recommendations. Studies scoring below 3/5 on the MMAT frequently lacked clear outcome definitions, appropriate comparison groups, or valid sexual function measurement tools, which limited their utility for this purpose.

We have now explicitly clarified this rationale in the Methods section and emphasized that quality assessment was used to enhance the trustworthiness of mapped intervention strategies, rather than to rank effectiveness or exclude evidence arbitrarily. We also acknowledge this approach as a deviation from conventional scoping review methodology and have transparently discussed it as a methodological consideration in the manuscript.

**Comment 5**

*“Very specific numbers… should not be applied to all surgeries… conclusions should be toned down.”*

**Response:**
We fully agree. The Results and Discussion sections have been revised to:

- Avoid generalization across surgical modalities,
- Remove language suggesting equivalence or comparability between surgical and non-surgical interventions.

Terms such as “robust” and “comparable” have been replaced with more cautious, evidence-aligned wording.

*Abstract, results:*

*The included 31 studies evaluated a broad range of interventions, including pelvic floor muscle training, behavioral therapies, pharmacological treatments, and surgical procedures. Reported effects on sexual function were inconsistent and varied across studies, depending on intervention type, study design, baseline sexual function status, and measurement tools. While some studies reported improvements in specific domains of sexual function following certain interventions, others reported no significant change or mixed outcomes. Evidence regarding partner-related sexual outcomes was limited and based on a small number of studies with modest sample sizes.*

*Abstract, conclusion:*

*This scoping review demonstrates that multiple interventions have been explored for their potential impact on sexual function in women with urinary incontinence; however, the available evidence is heterogeneous and largely descriptive. Due to variability in populations, interventions, and outcome assessment, no definitive conclusions regarding effectiveness or comparative benefit can be drawn. Future research using standardized sexual function measures and clearly defined populations is needed to better inform clinical practice and guideline development.*

*Discussion:*

*The review identified 31 studies. Improvements in sexual function outcomes were reported inconsistently across studies and varied by intervention type and outcome measure. Studies assessing pelvic floor muscle training (PFMT) demonstrated heterogeneous effects on sexual function. While some randomized controlled trials reported improvements in FSFI domains such as desire and satisfaction, other studies observed no statistically significant change following intervention. PFMT and PMS emerged as particularly robust, enhancing pelvic muscle tone and sexual satisfaction, while CBT addressed psychological barriers, improving sexual self-esteem [23, 25-28, 45]. Some studies about surgical interventions, including midurethral slings (MUS) and native tissue repairs, show enhanced sexual function and reduced coital incontinence, but some studies show sexual function deterioration[22, 23, 29-31, 35-40]. Non-surgical approaches were comparable to surgery for milder UI, offering less invasive options. However, interventions had minimal impact on partner sexual function, highlighting a patient-centric focus. These findings provide a comprehensive evidence base for tailored treatment strategies based on UI severity and patient preferences.*

**Comment 6**

*“The review uses the WHO definition of sexual health, but studies only measure sexual function.”*

**Response:**
We appreciate the reviewer’s thoughtful comment regarding the conceptual distinction between sexual health and sexual function. The decision to frame this review within the broader construct of sexual health was intentional and grounded in contemporary sexual and reproductive health theory.

From a sexual health perspective, urinary incontinence affects women not only through measurable physiological sexual function domains (e.g., desire, arousal, orgasm), but also through psychological well-being, sexual self-esteem, intimacy, relationship dynamics, and fear or avoidance of sexual activity. Although many included studies operationalized outcomes using validated sexual function instruments such as the FSFI and PISQ-12, these tools capture multiple dimensions that extend beyond isolated physiological function and reflect broader aspects of sexual health as defined by the World Health Organization.

Moreover, as this scoping review was designed as a foundational step toward informing future sexual health–oriented guidance, adopting a sexual health framework allowed us to situate sexual function outcomes within a more comprehensive and clinically meaningful context. Nevertheless, to avoid conceptual ambiguity, we have now explicitly clarified throughout the manuscript that the empirical outcomes synthesized in this review relate primarily to sexual function measures, while the overarching conceptual framework remains grounded in sexual health.

*Although sexual health is a multidimensional construct encompassing physical, psychological, and relational aspects of sexuality, the outcomes synthesized in this review primarily reflect sexual function as measured by validated questionnaires, which are commonly used proxies for sexual health–related outcomes in clinical research.*

**Comment 7**

*“Partner outcomes show minimal change, but only 2–3 small studies measured partner sexual function.”*

**Response:**
We agree and have revised the manuscript to:

- Clearly state that evidence on partner outcomes is very limited and exploratory.

*Finding:*

*Only three studies assessed partner-related sexual outcomes following interventions for urinary incontinence. Across these studies, with partner sample sizes that were either small and evaluated using heterogeneous outcome measures. Due to the limited number of studies, small sample sizes, and variability in outcome assessment, findings related to partner sexual outcomes should be interpreted with caution*

*.*

**Comment 8**

*“The paper says ‘no data available,’ but scoping reviews normally share extraction tables.”*

**Response:**
*We appreciate this clarification. We have:*

*Revised the Data Availability Statement to ensure internal consistency.*

*Full data extraction was presented in table as Supplementary File S1, detailing study characteristics and outcomes.*

**Minor Comments**

All typographical errors, inconsistent abbreviations (e.g., MUS vs. MUT), and figure quality issues have been corrected. Figures were remade following PLOS ONE technical guidelines, and recent RCTs (2020–2024) were added where relevant.

**Responses to Reviewer #2**

**Introduction**

*“The introduction is lengthy… the aim is not specific.”*

**Response:**
We have streamlined the Introduction and aligned the stated aim with the research question presented in the Methods section. The revised aim now clearly reflects the scoping purpose of mapping evidence and identifying gaps.

***Introduction*** *Urinary incontinence (UI) is a common and distressing condition defined by the International Urogynecological Association as any involuntary leakage of urine resulting in social or health-related discomfort [1]. Although it affects both sexes, UI is more prevalent among women due to anatomical, hormonal, and obstetric factors. Global prevalence estimates range from 3% to 55% and increase substantially with age [2].*

*UI is classified into stress urinary incontinence (SUI), urgency urinary incontinence (UUI), mixed urinary incontinence (MUI), and overflow incontinence. SUI involves leakage during increased intra-abdominal pressure and is commonly associated with urethral hypermobility or intrinsic sphincter deficiency, whereas UUI is characterized by involuntary leakage accompanied by urgency and is often linked to detrusor overactivity [3,4]. MUI presents features of both, while overflow incontinence, the least common subtype in women, results from bladder overdistension due to obstruction or neurological impairment [5].*

*UI represents a significant public health concern because of its high prevalence, economic burden, and negative impact on physical and psychological well-being [6]. Increasing evidence suggests that UI is associated with female sexual dysfunction (FSD), likely due to the close anatomical and functional relationship between the urinary and reproductive systems. However, research examining this association remains limited and methodologically heterogeneous, resulting in inconsistent findings [7]. Coital incontinence, in particular, is a highly distressing symptom that substantially impairs sexual satisfaction and intimacy [8].*

*Beyond physical symptoms, UI is associated with adverse mental health outcomes, including depressive symptoms, reduced self-esteem, and social withdrawal [9,10]. These multidimensional consequences underscore the importance of incorporating sexual health considerations into patient-centered UI care [11]. Current management strategies include conservative, pharmacological, and surgical approaches [12]; however, sexual health outcomes are not consistently evaluated when assessing treatment effectiveness [13].*

*Despite growing recognition of the relationship between UI and sexual health, substantial gaps persist in sexual health service provision for affected women. Healthcare providers frequently report limited knowledge or discomfort in addressing sexual concerns related to UI, contributing to stigma and unmet needs [14]. These challenges are amplified in conservative cultural settings where discussions of sexual health are particularly sensitive [15,16]. Furthermore, there is a lack of structured, evidence-based strategies specifically aimed at improving sexual health outcomes in women with UI [15].*

*Existing literature has largely focused on UI symptom management, with comparatively little attention given to interventions targeting sexual well-being [17]. Previous systematic reviews, including that by Pinheiro Sobreira Bezerra et al. (2020), have primarily examined prevalence and associations between UI and sexual function rather than systematically mapping intervention strategies [7].*

*The aim of this scoping review was to systematically map and describe the available evidence on surgical and non-surgical interventions evaluated for improving sexual health–related outcomes, primarily sexual function, in women with urinary incontinence, and to identify key knowledge gaps, including the limited evidence on partner-related sexual outcomes.*

**Methods / Results**

*“: While the methodology follows established guidelines, it fails to provide essential details on how sexual dysfunction was determined. It is not clear if the women in these studies had sexual dysfunction at baseline, which is a major limitation—it is difficult to study the improvement of a condition if the prevalence is unknown at the start. Additionally, while the authors mention using questionnaires like the PISQ and FSFI, they do not explain how these were used to draw conclusions or what was considered a "significant" change, especially in cases where no baseline dysfunction was recorded.*

*Additionally, regarding methodology, a major concern I have is grouping all Stress UI, Urge UI, and Pelvic Organ Prolapse together. These are biologically different conditions with unique treatment algorithms, and grouping them makes the results misleading. Because the surgical interventions mentioned are largely composed of prolapse repairs, they do not accurately represent UI treatment as a standalone category. This makes the comparison between surgical and non-surgical treatments invalid. On a positive note, the attempt to investigate partner satisfaction was interesting; the lack of significant reporting in this area serves as a helpful insight that more research is needed in this specific gap."*

**Response:**
We sincerely thank the reviewer for this detailed and clinically insightful comment. As specialists in sexual and reproductive health, we fully acknowledge the importance of conceptual clarity regarding sexual dysfunction assessment and population definition.

First, regarding the determination of sexual dysfunction, we agree that baseline sexual dysfunction status was inconsistently reported across the included studies, which represents a key limitation. Importantly, the primary aim of this scoping review was not to evaluate treatment effectiveness for diagnosed sexual dysfunction, but rather to map how sexual function–related outcomes have been assessed and reported following interventions for urinary incontinence. As such, inclusion was not restricted to studies enrolling women with confirmed sexual dysfunction at baseline. Instead, changes in sexual function scores (e.g., FSFI, PISQ-12 domains) were extracted and descriptively synthesized where reported. We have now explicitly clarified in the Methods and Limitations sections that improvements reflect reported changes in sexual function measures, rather than recovery from clinically defined sexual dysfunction.

Second, with respect to the use of sexual function questionnaires, we agree that the manuscript required greater transparency. The FSFI, PISQ, and related instruments were used in the included studies as validated tools to capture multidimensional aspects of sexual function. However, thresholds for “clinically significant” change were rarely predefined by primary studies. Therefore, our synthesis was intentionally descriptive and avoided applying uniform cut-off points or inferring clinical significance. We have now revised the Methods and Results sections to clarify that no standardized definition of meaningful change was imposed and that findings were interpreted cautiously.

Third, we appreciate the reviewer’s important concern regarding population heterogeneity. Stress UI, urgency UI, and pelvic organ prolapse are biologically distinct conditions with different treatment pathways. In the initial version, these conditions were grouped due to overlapping symptomatology and the way they were reported in primary studies. However, we fully agree that this approach may lead to misleading interpretations—particularly given that many surgical interventions primarily involved prolapse repair rather than UI-specific procedures. In response, we have explicitly stated that comparisons between surgical and non-surgical interventions should be interpreted with caution due to underlying population differences.

Finally, we appreciate the reviewer’s positive comment regarding partner satisfaction. We agree that the limited reporting of partner outcomes highlights an important evidence gap. This has now been emphasized more clearly in both the Results and Limitations sections as an area requiring further investigation.

*Method:*

*Sexual function outcomes were extracted as reported in the primary studies using validated questionnaires such as the Female Sexual Function Index (FSFI) and the Pelvic Organ Prolapse/Urinary Incontinence Sexual Questionnaire (PISQ). Baseline sexual dysfunction status was not consistently reported across studies; therefore, changes in sexual function scores were descriptively synthesized without assuming the presence of clinically diagnosed sexual dysfunction at study entry. No uniform thresholds for clinically significant change were applied, as these were rarely defined in the original studies.*

*Results:*

*Where data permitted, findings were stratified according to underlying condition (stress urinary incontinence, urgency urinary incontinence, mixed urinary incontinence, or pelvic organ prolapse).*

*Reported changes in sexual function scores reflect study-specific findings and should not be interpreted as treatment effects for established sexual dysfunction.*

*Limitation:*

*Interpretation of findings is limited by substantial population heterogeneity. Stress urinary incontinence, urgency urinary incontinence, and pelvic organ prolapse represent biologically distinct conditions with different pathophysiological mechanisms and treatment algorithms. As many surgical studies focused primarily on prolapse repair, comparisons between surgical and non-surgical interventions should be interpreted cautiously and not as direct alternatives for urinary incontinence management.*

**Discussion**

"The discussion claims that the findings have significant implications for clinical practice, but this feels like an overstatement that "oversells" the outcome. Overall, the review does not add much to the existing knowledge because its heterogeneous nature—including women with and without sexual dysfunction across different types of UI—makes it difficult to accurately assess the impact of treatment."

**Response:**
We appreciate the reviewer’s thoughtful critique and agree that the initial wording may have overstated the direct clinical applicability of the findings. We have revised the Discussion to avoid implying practice-changing implications and to better reflect the descriptive and exploratory nature of this scoping review.

Importantly, the contribution of this review is not positioned as establishing treatment effectiveness, but rather as clarifying the current state and limitations of the evidence base regarding sexual health–related outcomes in women with urinary incontinence. From a sexual health perspective, heterogeneity in populations, baseline sexual function status, and outcome assessment is not merely a methodological weakness but a central finding that explains why clear clinical guidance in this area remains lacking.

By systematically mapping how sexual function outcomes have been evaluated across diverse interventions and clinical contexts, this review contributes to existing knowledge by:

1. Identifying the conceptual conflation between urinary incontinence treatment and sexual health outcomes in current research;
2. Demonstrating the lack of standardized baseline assessment of sexual dysfunction and clinically meaningful change;
3. Highlighting the inconsistency in outcome measurement and reporting, which limits comparability across studies; and
4. Exposing critical evidence gaps, particularly regarding partner-related outcomes.

Therefore, while heterogeneity limits causal inference, it does not negate the scientific value of the review. Instead, it provides essential insight into why evidence remains fragmented and underscores the need for more methodologically rigorous, sexual health–informed research. We have revised the Discussion to emphasize these contributions and to frame clinical implications cautiously, focusing on research and service development rather than immediate changes to clinical practice.

*Discussion:*

*The review identified 31 studies. Improvements in sexual function outcomes were reported inconsistently across studies and varied by intervention type and outcome measure. The heterogeneity observed across populations, interventions, and outcome measures should not be interpreted as a lack of scientific contribution. Instead, it reflects the current state of sexual health research in the context of urinary incontinence, where sexual function is often treated as a secondary or poorly defined outcome. By systematically documenting this heterogeneity, the present review clarifies why existing evidence fails to support definitive clinical conclusions and identifies specific methodological priorities for future research*…….

*Rather than providing practice-changing guidance, the findings of this scoping review highlight important gaps and inconsistencies that currently limit the translation of sexual health outcomes into routine urinary incontinence care, emphasizing the need to integrate sexual health assessments into routine UI management. Clinicians may use e non-surgical interventions such as PFMT and PMS as first-line treatments for women with mild to moderate UI, given their safety and patient acceptability. For severe cases, surgical options like MUT offer durable benefits but require careful patient selection and preoperative counseling to mitigate risks of sexual dysfunction. The limited impact on partner sexual function suggests that clinicians can explore couple-based interventions to address relational dynamics, potentially incorporating CBT to enhance intimacy and communication.*

*From a clinical sexual health perspective, the primary implication of these findings is the need for more structured and consistent assessment of sexual health in women with urinary incontinence, rather than immediate modification of treatment algorithms.*

We sincerely thank the Editor and Reviewers for their valuable feedback. We believe the revised manuscript is substantially strengthened and hope it will now be suitable for publication in PLOS ONE.

Kind regards,

Dr. Shahali
(on behalf of all authors)
